# Formation of bimetallic clusters in superfluid helium nanodroplets analysed by atomic resolution electron tomography

**DOI:** 10.1038/ncomms9779

**Published:** 2015-10-28

**Authors:** Georg Haberfehlner, Philipp Thaler, Daniel Knez, Alexander Volk, Ferdinand Hofer, Wolfgang E. Ernst, Gerald Kothleitner

**Affiliations:** 1 Graz Centre for Electron Microscopy, Steyrergasse 17, 8010 Graz, Austria; 2 Institute for Electron Microscopy and Nanoanalysis, Graz University of Technology, Steyrergasse 17, 8010 Graz, Austria; 3 Institute of Experimental Physics, Graz University of Technology, Petersgasse 16, 8010 Graz, Austria

## Abstract

Structure, shape and composition are the basic parameters responsible for properties of nanoscale materials, distinguishing them from their bulk counterparts. To reveal these in three dimensions at the nanoscale, electron tomography is a powerful tool. Advancing electron tomography to atomic resolution in an aberration-corrected transmission electron microscope remains challenging and has been demonstrated only a few times using strong constraints or extensive filtering. Here we demonstrate atomic resolution electron tomography on silver/gold core/shell nanoclusters grown in superfluid helium nanodroplets. We reveal morphology and composition of a cluster identifying gold- and silver-rich regions in three dimensions and we estimate atomic positions without using any prior information and with minimal filtering. The ability to get full three-dimensional information down to the atomic scale allows understanding the growth and deposition process of the nanoclusters and demonstrates an approach that may be generally applicable to all types of nanoscale materials.

## Abstract (web summary)

Advancing electron tomography to atomic resolution is a powerful and challenging process. Here, the authors demonstrate atomic resolution electron tomography on silver-gold core-shell nanoclusters grown in superfluid helium nanodroplets, revealing their three-dimensional morphology and composition.

## Graphical abstract

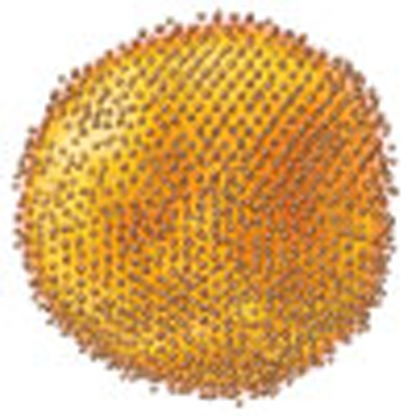

Metallic nanoparticles consisting of a few thousand atoms are of large interest for potential applications in different fields such as optics^1^, catalysis^2^ or magnetism^3^. Superfluid helium droplets represent a versatile, novel tool for designing such nanoparticles, allowing fine-tuned synthesis of pure or composite clusters for a wide range of materials^4^,^5^,^6^. Using ultra-high vacuum conditions and getting on without solvents or additives compared with chemical synthesis, the method delivers high purity materials, which can be well controlled in terms of size and composition. In this paper, we investigate core-shell nanoparticles produced using the He-droplet technique. We analyse their structure, composition and morphology at the atomic level by advancing electron tomographic methods to better understand and optimize their unique properties.

Despite significant recent progress in three-dimensional (3D) electron tomographic imaging^7^,^8^,^9^,^10^,^11^, the ultimate goal of resolving position and type of each single atom inside a material remains elusive. First reconstructions at atomic resolution were demonstrated for monocrystalline particles from few images taken along high-index crystallographic orientations, applying strong constraints in the reconstruction algorithms^12^,^13^,^14^. For polycrystalline or amorphous materials, such approaches are not applicable and a full tomographic tilt series needs to be carried out. Near-atomic resolution of single-metallic polycrystalline particles has been first demonstrated on gold nanoparticles^15^, and more recently Chen *et al*.^16^ have mapped 3D dislocations in platinum nanoparticles at atomic resolution using very strong filtering in the Fourier domain. This approach has been used to some success, but has also led to some controversy^17^ and, moreover, is not necessary when careful considerations in the reconstruction are applied to low-noise experimental data. Extending atomic resolution electron tomography to composite materials such as bimetallic polycrystalline nanoparticles, by locating (structure) as well as identifying (composition) atoms in all three dimensions, is even more challenging. For this, we adapt the image acquisition scheme to obtain high-resolution projections with improved signal-to-noise ratio and minimal distortions. Without any prior assumption about the structure, without a particular tilt scheme or special filtering, we reconstruct the nanoparticle applying rather standard reconstruction algorithms. Demonstrated for a Au/Ag bimetallic system, we reveal Au- and Ag-rich regions and we estimate atomic positions. We then use this information to answer fundamental questions about the growth of nanoclusters in superfluid helium droplets.

## Results

### Nanocluster growth

**Figure 1:**
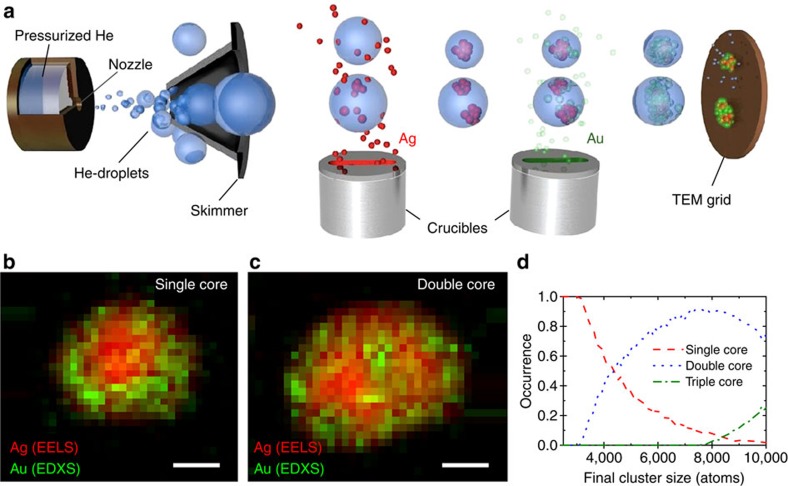
Synthesis and characterization of single- and multi-core nanoclusters. (**a**) Schematic of the experimental setup for cluster synthesis: superfluid He-droplets are guided through two doping cells and terminated on a TEM grid. (**b**,**c**) Elemental maps of a single-core/shell Ag-Au cluster (**b**) and of a dual-core/shell cluster (**c**). The Ag elemental maps are extracted from the EELS signal of the Ag M-edge, the Au elemental maps from the Au L-lines in the energy-dispersive x-ray spectroscopy (EDXS) signal. (**d**) Calculated occurrence probability of single-, double- and triple-core clusters as a function of final cluster size. Scale bars, 2 nm.

A beam of superfluid helium droplets (*T*=0.4 K)^18^ is guided through two doping cells containing vapours of the desired dopants (Fig. 1a). By passing through the vapour, the He droplets pick up foreign atoms. We achieve a Ag-Au core-shell structure by adding Ag to the droplet first and Au second in sequential doping cells^6^. For transmission electron microscopy (TEM) observation, the droplet beam is terminated on an amorphous carbon TEM grid, where the helium evaporates while the dopant is adsorbed. The low impact velocity of ∼200 m s^−1^ as well as the helium, acting as a cushion, ensure so-called soft landing, limiting structural modification during deposition. The size distribution of the clusters was ranging between 2 and 8 nm (Supplementary Fig. 1), governed by the size distribution of the He droplets^19^.

### Single- and multi-centre growth of core-shell nanoclusters

Analytical TEM investigations reveal smaller clusters mainly consisting of a single silver core, surrounded by a gold shell, whereas larger clusters contain two or more silver grains embedded in a gold matrix (Fig. 1b, c and Supplementary Fig. 2). The largest diameters of single-core clusters were measured to be 5 nm, whereas the smallest double core clusters amounted to 7 nm. With the transition threshold between a single- and double-core set between these numbers, a cluster size of ∼5,000 atoms (2,500 Ag/Au atoms) or an initial droplet size of 5 × 10^7^ He atoms can be obtained as a crossover value. Based on a simple estimation of the recombination time for two atoms inside a helium droplet^20^, a transition from single-centre growth to multi-centre growth has been predicted at an initial droplet size of 5 × 10^5^ He atoms. Applying more elaborate *ab-initio* simulation data^21^ as a model, one can calculate the entire growth behaviour of clusters inside a droplet (Methods). Thereby, newly added dopant atoms aggregate very fast, keeping the total number of clusters inside the droplet low. At the droplet size where multi-centre aggregation starts (5 × 10^5^ He atoms), initially separated clusters will aggregate very shortly after the droplet leaves the first pickup cell. In sufficiently large droplets, however, two or more separated clusters of the first species can survive until they arrive at the second pickup cell. Using our experimental parameters in this model (Fig. 1d), we find the crossover between single- and double-core clusters at 4,000–5,000 atoms (initial droplet size: 4–5 × 10^7^ He atoms), matching well with the experimental value and supporting our model for multi-centre aggregation. The experimental parameters and setup can be used to tune the mean cluster size and composition^19^ to favour either single- or multi-core clustering and thereby customize the clusters' properties, such as their optical response^20^. In even larger droplets than investigated here, gold and silver can potentially condensate independently inside the droplet^6^.

### 3D analysis and atomic position estimation

**Figure 2:**
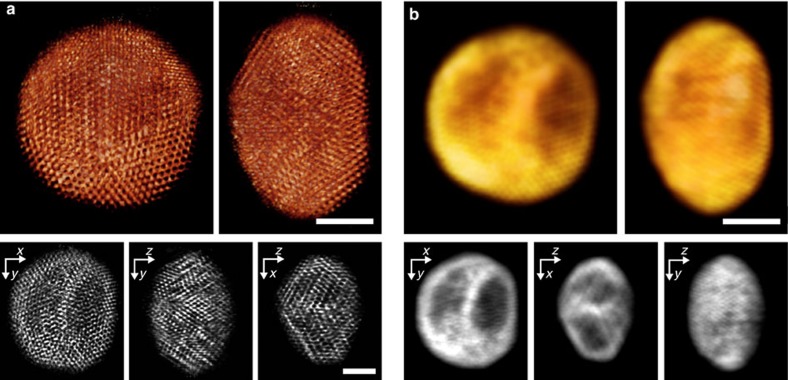
3D reconstructions of a Ag-Au nanoclusters. Reconstruction showing structure (**a**) and composition (**b**) of the cluster. For each reconstruction, a volume-rendered 3D view and three orthogonal 0.23 Å-thick slices through the reconstruction are shown. Scale bars, 2 nm.

A single cluster was analysed three-dimensionally for its atomic structure, shape and composition. Thirty-one high-angle annular dark field scanning TEM (STEM) projections were taken between −72° and +70°, optimized for signal-to-noise, by adding up 15 drift-corrected, fast-scan images at each tilt (Methods and Supplementary Fig. 3). The tilt series, aligned with a centre of mass approach, was then subject to both a multiplicative SIRT^22^ (simultaneous iterative reconstruction technique) and a TV^10^,^23^ (total-variation) minimization algorithm. In the SIRT reconstruction (Fig. 2a and Supplementary Movie 1), fivefold symmetry centres are visible together with darker and brighter regions, which can be attributed to silver- or gold-rich regions. This comes out even clearer in the TV reconstructed model, where only composition information is kept (Fig. 2b and Supplementary Movie 2). As expected for this cluster size, two silver cores are visible as darker regions, separated by gold with a minimal thickness of 2–3 atomic layers. This double core structure was also confirmed by 2D electron energy-loss spectroscopy (EELS) measurements on the same particle (Supplementary Fig. 4).

**Figure 3:**
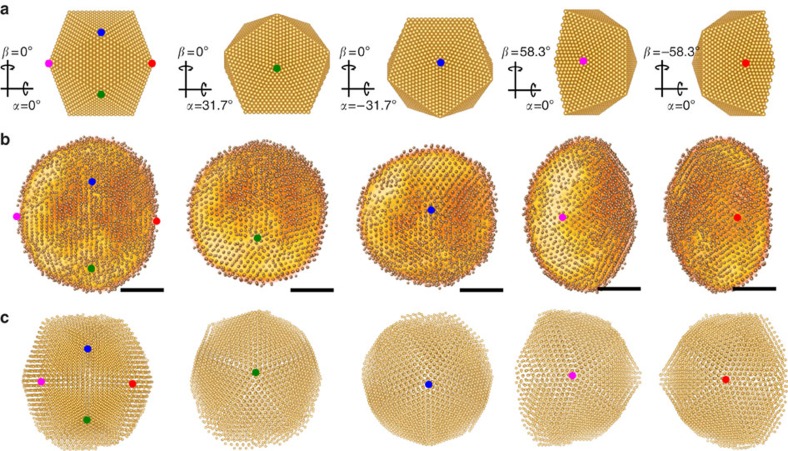
Model, reconstruction and simulation of atomic positions in the nanocluster. (**a**) Model of a modified icosaheder (lower part cutoff) seen from the top and along four different fivefold symmetry axes. (**b**) Reconstruction of atomic positions seen along the same directions. (**c**) Result of molecular dynamics simulation of a soft-landed cluster on a carbon film seen along the same directions. Fivefold symmetry centres are indicated in all images. Scale bars, 2 nm.

By searching for confined maxima in the SIRT reconstruction (Methods and Supplementary Fig. 5), 7,150 atomic positions can be localized within the cluster volume. Measured to be 119 nm^3^, a 4.08^3^ Å^3^ unit cell in the fcc lattice would correspond to 7,050 atoms, matching approximately the experimental value. The cluster appears in a highly symmetric multiply twinned structure (overlaid with the compositional reconstruction, Fig. 3 and Supplementary Movie 3), shaped roughly as an icosahedron, which is structurally modified due to binding of the cluster to the surface. In the figure, both a model of a modified icosahedron (Fig. 3a) and the reconstruction (Fig. 3b) are tilted in the same way to centre different fivefold symmetry axes (cf. Supplementary Fig. 6). Although fivefold symmetries can be also observed in 2D projections, only the tomographic reconstruction revealed modifications of the icosahedral structure because of surface binding.

## Discussion

3D information from the final state of the cluster on the substrate can now be linked to the growth and deposition process. From simulations^24^ and considerations of the binding energy^25^, it is known that high temperatures or even partial melting during cluster–cluster collisions within the helium droplet and deposition on the substrate can occur on a very short timescale (few picoseconds). The fact, however, that the double core structure survives aggregation and deposition, and does not form an alloy as expected in a melt^26^, implies efficient cooling by the matrix and restricts melting processes to the contact region. Furthermore, a molten cluster of this size would cool from the outside and will not adopt this highly symmetric multiply twinned form^27^. Efficient cooling is guaranteed if the He remains liquid around the clusters and does not form a gaseous layer that would reduce heat dissipation. This condition is still fulfilled for the present cluster size but this could change for even larger clusters^25^. We can also observe that the structure of the nanoparticle does not change significantly between Au-rich and Ag-rich regions. This indicates that due to the similar lattice constants of Ag and Au, the different types of atoms arrange themselves on a common lattice and that the different phases do not affect the structure of the cluster.

Considering the last steps of the aggregation process (cluster–cluster aggregation) one would expect rather irregular cluster shapes at the time of landing. The tomographic reconstruction shows a smooth lenticular shape of the cluster although. Its size is about 8 × 7 × 5 nm^3^, with the smallest dimension being in the direction perpendicular to the substrate. Given a significant amount of time (few hours) passed between deposition of the clusters and the imaging, the smooth shape of the cluster likely stems from surface diffusion; a mechanism that preserves the inner structure but relaxes the outer shape of the cluster.

Simulations for smaller clusters predict a lenticular shape because of the binding of the cluster to the surface^24^. Following this approach, the landing of an icosahedral cluster on an amorphous carbon substrate was simulated using molecular dynamics (see Supplementary Movie 4 for details) and the final shape of the cluster has been compared with the reconstruction (Fig. 3c). Even though we do not expect an ideal icosahedral shape as starting condition, the deformation of the cluster owing to binding to the substrate and the impact of landing on its inner structure can be explored; for these two parameters (aspect ratio and inner structure), we observe good agreement between experiment and simulation. The exterior cluster shape comes out differently although, which is anticipated because of the small amount of time, framed by the simulation. The simulation stops one nanosecond after the landing of the cluster, whereas relaxation to the equilibrium shape because of surface diffusion may take minutes or even hours^27^. Even though multiply twinned clusters do not represent the minimum energy configuration for clusters larger than a few hundred atoms^28^, fivefold symmetric substructures are not rare when growing clusters. Still, the high symmetry of the icosahedral structure in such a large cluster would be very surprising without relaxation effects, supporting the interpretation that surface diffusion leads to the relaxation of the outer shell of the soft-landed cluster, which then adopts the symmetry of the inner region.

In conclusion, we have estimated atomic positions within nanoparticles in 3D without any prior information, while getting at the same time information about the local elemental composition at near-atomic resolution. This approach was used to get insights into the growth and deposition process of composite nanoclusters created in superfluid helium droplets, revealing the mechanisms responsible for particle structure and for single- or multi-core particle formation. This understanding will allow fine-tuning of process parameters to optimize properties of nanoclusters for applications in optics, catalysis and other fields. This work establishes electron tomography as a singularly powerful tool for analysing complex nanostructures at 3D atomic resolution, from composite nanoparticles to nanowires or quantum dots. Furthermore, there is potential to approach true elemental identification at the atomic level using EELS or EDXS tomography.

## Methods

### Particle synthesis

The experimental setup used to produce the core-shell clusters for this investigation consists of three vacuum chambers with subsequently decreasing baseline pressure (*p*_0_). In the first chamber, a beam of helium droplets (He_N_) is produced by expanding pressurized (*p*_nozzle_=20 bar), high purity He (99.9999%) through a 5-μm nozzle, cooled to cryogenic temperatures (*T*_nozzle_=8 K) into high vacuum (*p*_0_=10^−5^ mbar). During this free jet expansion, the He cools down even further and superfluid droplets are formed, which maintain a temperature of ∼0.4 K by evaporative cooling^18^. Placed a few millimetres after the nozzle, a skimmer is shaping the droplet beam, which continues into the second chamber (*p*_0_=10^−7^ mbar), where it is guided through two metal vapour cells. In these cells, resistively heated crucibles generate a vapour of the desired dopant materials, whose pressure is tunable by temperature variation. As the beam passes through the cells, droplets colliding with dopant atoms pick up the foreign species with a probability close to unity. This way up to several thousand atoms are collected along the trajectory of a droplet. Inside the He_N_ the dopant atoms agglomerate and form clusters. Employing a sequential pickup scheme, Ag-Au core-shell clusters can be formed^6^. The binding energy released during the cluster formation process is dissipated by the droplet and leads to the evaporation of roughly 1,600 He atoms per electron-volt binding energy. In the case of large metal clusters, ∼5,000 He atoms are evaporated per dopant atom, leading to a significant shrinking of the droplet. The relative decrease of He in the beam can be taken as a measure to adjust the composition of the clusters. After the doping in the second chamber, the droplet beam passes through another skimmer and enters the last chamber (*p*_0_=10^−10^ mbar) where the beam is terminated on a commercial TEM substrate (Ted Pella Inc.). It consists of an ultra-thin amorphous carbon (a-C) film with a thickness <3 nm, backed with a holey carbon support film on a 400 mesh copper grid. During the landing process, the He evaporates, and cushions the impact. This and the low velocity of the beam (∼ 200 m s^−1^) lead to so-called soft landing, which avoids major structural changes upon deposition. For the transport to the microscope, the samples were shortly exposed to ambient conditions (∼1 min). Vacuum transfer would be preferable, however, at the moment vacuum transfer sample holders, compatible with tomography are not available. Au and Ag nanoparticles, stored several days under ambient conditions, have shown no visible signs of oxidation.

### Simulation of cluster growth

As a detailed simulation of the aggregation process inside the droplets is computationally very costly, a simplified calculation was conducted to gain insight into the process. From experiments, the size and velocity of the helium droplets as well as the size of the clusters produced from a specific droplet size are known. With this and the geometry of our pickup-source, the rate at which dopant atoms are added to the droplet can be calculated and compared with the timescales for aggregation. The doping process is modelled by spawning single atoms with thermal velocity (8 m s^−1^ at 0.4 K for Ag-atoms) at the known doping frequency. The thermal velocity was chosen over the Landau velocity, as in our case, we do not assume the matrix to stay in the superfluid state at all times because of strong exothermic reactions taking place in the helium environment. It is assumed that every two-particle collision leads to the formation of a cluster, containing all atoms of the colliding particles. The time it takes for two atoms to collide was taken from Hauser *et al*.^21^ For the collision of clusters, the collision times were corrected based on the lower velocity of the aggregates, considering conservation of momentum. If more than two particles are present in the droplet, the probability of a collision grows as the number of pairs between these particles. The binding energy released upon aggregation is dissipated by the surrounding He-matrix, leading to a shrinking of the droplet. This reduces the accessible volume and therefore the time between collisions. In this model, only a small number (few tens) of clusters are present in a droplet at any time. During the whole doping process, faster (smaller) clusters aggregate after a very short period of time. By contrast, larger clusters inside the droplet can survive for a significant period of time, even after the droplet leaves the doping cell. In the experiment, where a sequential pickup scheme is employed, large droplets can contain more than one cluster of the first species as they arrive at the second pickup-cell. Employing our experimental parameters, we run calculations for several cluster sizes up to 10,000 atoms. The crossover between single-core and double-core configurations occurs at 4,000–5,000 atoms (2,000–2,500 of each species), where half of the particles features one core and the second half has two cores, when reaching the second doping cell (Fig. 1d).

### Simulation of cluster landing

To simulate landing of a cluster on an amorphous carbon substrate. molecular dynamics simulations of the cluster deposition process and the substrate interaction were carried out. For the cluster, a model with icosahedral morphology comprised of 6,525 gold atoms was used. The integration of Newton's equations of motion was performed by using a Velocity Verlet algorithm. For temperature control, we used an Andersen thermostat with a collision frequency of 2 THz. To reduce perturbation of the system, only atoms next to the substrate were considered by the thermostat. The interatomic forces were calculated by using a semi-empirical many-body potential proposed by Sutton and Chen^29^. For the interaction with the amorphous carbon support, a parameterized Lennard-Jones 6–12 potential obtained from *ab-initio* calculations^30^ was applied.

Initially, the cluster was thermalized at 0.4 K. Throughout the landing process, the time step was set to 5 fs and increased after 50 ps to 10 fs, during thermalization to the substrate temperature of 300 K. After 250 ps, the system is fully thermalized and finally evolved for 1 ns with a time step of 20 fs.

### Experimental crossover between single- and double-core growth

For an estimation of the crossover between single- and double-centre aggregation, we analysed the elemental maps shown in Supplementary Fig. 2. The largest single-core clusters had a size of 5 nm in the direction parallel to the substrate and 4.5 nm in the vertical image direction (under 65° tilt angle). The smallest double-core clusters had a diameter of 7 nm parallel to the substrate and 5 nm in the vertical image direction. Putting the crossover in between these values, we calculated the volume for an ellipsoid with a size of 6 nm for two principal axes and 4.5 nm for the third principal axis resulting in a volume of 85 nm^3^. This corresponded to 5,000 atoms in the fcc-lattice with a unit cell size of 4.08^3^ Å^3^.

### STEM EELS and EDXS

For all TEM experiments, a C_S_-probe-corrected microscope (FEI Titan^3^ 60-300) equipped with an imaging filter (Gatan GIF Quantum) and a four-quadrant EDX detector (FEI Super-X) was used. The microscope was operated in scanning mode at 300 kV with the electron beam set to a current of ∼130 pA and a convergence semi-angle of 19.6 mrad. EDXS spectra and dual-EELS spectra were acquired simultaneously into spectrum images, by means of the DigiScan engine in the Gatan Microscopy Suite. EDXS spectra were accumulated for a pixel time of 50 ms and the EELS spectra were acquired with a collection semi-angle of 20.5 mrad and a dispersion of 0.25 eV per pixel. The pixel time was 50 μs for the low-loss spectra (from −50 to 450 eV) and 50 ms for the core-loss spectra (from 150 to 650 eV). All analytical data except for Supplementary Fig. 5 were recorded at a tilt angle of 65° to minimize shadowing of the X-ray detectors from the sample holder (Fischione 2020 Advanced Tomography Holder).

Spectrum images have been drift-corrected after acquisition assuming a time-dependent linear drift model, as illustrated in Supplementary Fig. 7. The survey image, which was taken before acquisition of the spectrum image with a short dwell time, was taken as reference for the signal from the same detector recorded during spectrum image acquisition. A least-squares optimization was carried out in MATLAB, comparing the intensities from the survey image with the signal acquired during spectrum image acquisition to calculate drift parameters (drift direction: *α*, drift velocity: *v*_drift_, initial drift in *x*-direction: *x*_start_ and *y*-direction: *y*_start_). These parameters were subsequently used to compensate for drift in the spectrum images.

For the extraction of elemental maps from EDXS data, the background was modelled by Kramer's approach, and peak families were fitted by Gaussian models within the Gatan Microscopy Suite. For EELS analysis, the background was described with power-law function and the Ag M-edge as well as the C K-edge overlapping with the Ag M-edge, were modelled by Hartree–Slater ionization cross-sections. In addition, the low-loss spectrum was used to compensate for plural-scattering and for better modelling the near-edge fine structure.

### Tilt series acquisition

Tilt series were acquired between −72° and +70° with a linear tilt step of 5° from −70° to +70° and two additional projections at −72° and +68°. The tilt range was limited by the shadowing of the sample holder (Fischione 2020 Advanced Tomography Holder). At each tilt angle, focusing was done on a neighbouring particle. High-angle annular dark field STEM images were acquired with a convergence semi-angle of 19.6 mrad at a camera length of 91 mm (detector range: 57–200 mrad). Image size was 512 × 512 pixels at a pixel size of 0.23 × 0.23 Å^2^. To limit the effects of sample drift at each tilt angle, 15 images were acquired with a short dwell time of 2.4 μs per pixel. This was done automatically using a script in the Gatan Microscopy Suite. The 15 images were aligned and summed up using a routine in MATLAB. This alignment routine is illustrated in Supplementary Fig. 8. The shift in *x*- and *y*-direction of each image with respect to the first image was calculated by filtered cross-correlation. These shift values were then taken as shift parameters for the central pixel of each image. Based on the total acquisition time for the 15 images, the drift at each time instant was interpolated. With the timing parameters of the image acquisition (acquisition time and dwell time), the *x*- and *y*-shifts for each individual pixel could be retrieved and then used to compensate for the drift in each image. The 15 corrected images were summed up to form the projections at each tilt angle (Supplementary Fig. 7). Even though we observed minor restructuring on the surface of the particle during the experiment, the inner structure of the particles did not change and no annealing was observed.

### Tilt series alignment and background removal

Before the tilt series alignment, the background of each projection was removed. For this purpose, the mean grey level in the outer regions of the image, away from the cluster, was calculated for each projection and subtracted from each pixel. In this way, the signal contribution of the carbon film could be eliminated. Afterwards, a mask was created around the particle using thresholding and morphological operators. All pixels outside of this mask were set to 0.

For the alignment of the tilt series, centre of mass methods were employed. The centre of mass of each projection was calculated and used as new centre for the aligned projection. For the retrieval of the tilt axis, rotational centres for all sinograms are calculated based on centre of masses^31^ and a linear fit through all rotational centres of all sinograms enabled to determine the axis position.

### Tomographic reconstruction

For the tomographic reconstructions, two different types of algorithm have been used. First, a reconstruction was done using a multiplicative SIRT algorithm with 200 iterations and a relaxation parameter *λ*=0.3, implemented in MATLAB and based on projection and backprojection operators from the ASTRA toolbox using the Graphics Processing Unit^32^. With this reconstruction, high-resolution structural information (Fig. 2a) could be obtained. Comparison of original projections and re-projected data is shown in Supplementary Fig. 9a,b.

Second, a 3D TV minimization algorithm^10^ based on the solver TVAL3 (ref. 23) and Graphics Processing Unit-based projection and backprojection operators from the ASTRA toolbox was chosen to gain compositional information (Fig. 2b). A small weighting parameter (*μ*=2) was used, corresponding to a strong TV regularization of the reconstruction. This led to large constant regions in the reconstruction, effectively smoothing out structural information and only leaving compositional contrast. The comparison between original projections and re-projections in Supplementary Fig. 9 visualizes this effect also in the re-projections.

### Estimation of atomic positions

To locate atomic positions, the SIRT reconstruction was convolved in 3D with a Gaussian kernel with a full-width at half-maximum of 1.8 Å (Supplementary Fig. 5a,b). Thereby, high-frequency noise and artefacts could be minimized, yet maintaining enough resolution to identify the atomic structure. Within this volume, a search for local maxima was performed, comparing each voxel to its 26 neighbours. In a last step for maxima closer together than 1.4 Å, the lower maximum was removed. All the coordinates found this way were used as atomic positions and can be displayed as spheres, like in the slices through the volume in Supplementary Fig. 5c.

## Additional information

**How to cite this article:** Haberfehlner, G. *et al*. Formation of bimetallic clusters in superfluid helium nanodroplets analysed by atomic resolution electron tomography. *Nat. Commun.* 6:8779 doi: 10.1038/ncomms9779 (2015).

## Supplementary Material

Supplementary Figures Supplementary Figures 1-9

Supplementary Movie 1 3D reconstruction showing structure of the Ag-Au nanocluster

Supplementary Movie 2 3D reconstruction showing composition of the Ag-Au nanocluster

Supplementary Movie 3 Reconstruction of atomic positions within the nanocluster overlaid with compositional information

Supplementary Movie 4 Simulation of landing of a nanocluster on an amorphous carbon substrate

## References

[b1] Link S. & El-Sayed M. A. Optical properties and ultrafast dynamics of metallic nanocrystals. Annu. Rev. Phys. Chem. 54, 331–366 (2003). 12626731 10.1146/annurev.physchem.54.011002.103759

[b2] Heiz U. & Landman U. Nanocatalysis Springer Berlin Heidelberg (2007).

[b3] Bansmann J. . Magnetic and structural properties of isolated and assembled clusters. Surf. Sci. Rep. 56, 189–275 (2005).

[b4] Mozhayskiy V., Slipchenko M. N., Adamchuk V. K. & Vilesov A. F. Use of helium nanodroplets for assembly, transport, and surface deposition of large molecular and atomic clusters. J. Chem. Phys. 127, 094701 (2007). 17824753 10.1063/1.2759927

[b5] Boatwright A. . Helium droplets: a new route to nanoparticles. Faraday Discuss. 162, 113–124 (2013). 24015579 10.1039/c2fd20136d

[b6] Thaler P. . Formation of bimetallic core-shell nanowires along vortices in superfluid He nanodroplets. Phys. Rev. B 90, 155442 (2014).

[b7] Arslan I., Yates T. J. V., Browning N. D. & Midgley P. A. Embedded nanostructures revealed in three dimensions. Science 309, 2195–2198 (2005). 16195455 10.1126/science.1116745

[b8] Barnard J. S., Sharp J., Tong J. R. & Midgley P. A. High-resolution three-dimensional imaging of dislocations. Science 313, 319 (2006). 16857932 10.1126/science.1125783

[b9] Nicoletti O. . Three-dimensional imaging of localized surface plasmon resonances of metal nanoparticles. Nature 502, 80–84 (2013). 24091976 10.1038/nature12469

[b10] Haberfehlner G., Orthacker A., Albu M., Li J. & Kothleitner G. Nanoscale voxel spectroscopy by simultaneous EELS and EDS tomography. Nanoscale 6, 14563–14569 (2014). 25349984 10.1039/c4nr04553j

[b11] Midgley P. A. & Thomas J. M. Multi-dimensional electron microscopy. Angew. Chem. Int. Ed. 53, 8614–8617 (2014). 10.1002/anie.201400625 24919685

[b12] Van Aert S., Batenburg K. J., Rossell M. D., Erni R. & Van Tendeloo G. Three-dimensional atomic imaging of crystalline nanoparticles. Nature 470, 374–377 (2011). 21289625 10.1038/nature09741

[b13] Goris B. . Atomic-scale determination of surface facets in gold nanorods. Nat. Mater. 11, 930–935 (2012). 23085569 10.1038/nmat3462

[b14] Goris B. . Three-dimensional elemental mapping at the atomic scale in bimetallic nanocrystals. Nano Lett. 13, 4236–4241 (2013). 23952010 10.1021/nl401945b

[b15] Scott M. C. . Electron tomography at 2.4-angstrom resolution. Nature 483, 444–447 (2012). 22437612 10.1038/nature10934

[b16] Chen C.-C. . Three-dimensional imaging of dislocations in a nanoparticle at atomic resolution. Nature 496, 74–77 (2013). 23535594 10.1038/nature12009

[b17] Rez P. & Treacy M. M. J. Three-dimensional imaging of dislocations. Nature 503, E1–E1 (2013). 24256805 10.1038/nature12660

[b18] Callegari C. & Ernst W. E. in Handbook of High Resolution Spectroscopy Vol. 3, 1st edn (eds Merkt, F. & Quack, M.) 1551–1594 (John Wiley & Sons, Chichester (2011).

[b19] Toennies J. P. & Vilesov A. F. Superfluid helium droplets: a uniquely cold nanomatrix for molecules and molecular complexes. Angew. Chem. Int. Ed. 43, 2622–2648 (2004). 10.1002/anie.200300611 18629978

[b20] Loginov E. . Photoabsorption of AgN(N∼6–6000) nanoclusters formed in helium droplets: transition from compact to multicenter aggregation. Phys. Rev. Lett. 106, 233401 (2011). 21770503 10.1103/PhysRevLett.106.233401

[b21] Hauser A. W., Volk A., Thaler P. & Ernst W. E. Atomic collisions in suprafluid helium-nanodroplets: timescales for metal-cluster formation derived from He-density functional theory. Phys. Chem. Chem. Phys. 17, 10805–10812 (2015). 25812719 10.1039/c5cp01110h PMC4441260

[b22] Gilbert P. Iterative methods for the three-dimensional reconstruction of an object from projections. J. Theor. Biol. 36, 105–117 (1972). 5070894 10.1016/0022-5193(72)90180-4

[b23] Li C., Yin W., Jiang H. & Zhang Y. An efficient augmented Lagrangian method with applications to total variation minimization. Comput. Optim. Appl. 56, 507–530 (2013).

[b24] Thaler P., Volk A., Ratschek M., Koch M. & Ernst W. E. Molecular dynamics simulation of the deposition process of cold Ag-clusters under different landing conditions. J. Chem. Phys. 140, 044326 (2014). 25669542 10.1063/1.4862917

[b25] Gordon E. B., Karabulin A. V., Matyushenko V. I., Sizov V. D. & Khodos I. I. The role of vortices in the process of impurity nanoparticles coalescence in liquid helium. Chem. Phys. Lett. 519–520, 64–68 (2012).

[b26] Link S., Wang Z. L. & El-Sayed M. A. Alloy formation of gold−silver nanoparticles and the dependence of the plasmon absorption on their composition. J. Phys. Chem. B 103, 3529–3533 (1999).

[b27] Henry C. R. Morphology of supported nanoparticles. Prog. Surf. Sci. 80, 92–116 (2005).

[b28] Baletto F. & Ferrando R. Structural properties of nanoclusters: energetic, thermodynamic, and kinetic effects. Rev. Mod. Phys. 77, 371–423 (2005).

[b29] Sutton A. P. & Chen J. Long-range Finnis–Sinclair potentials. Philos. Mag. Lett. 61, 139–146 (1990).

[b30] Werner R., Wanner M., Schneider G. & Gerthsen D. Island formation and dynamics of gold clusters on amorphous carbon films. Phys. Rev. B 72, 045426 (2005).

[b31] Azevedo S. G., Schneberk D. J., Fitch J. & Martz H. E. Calculation of the rotational centers in computed tomography sinograms. IEEE Trans. Nucl. Sci. 37, 1525–1540 (1990).

[b32] Palenstijn W. J., Batenburg K. J. & Sijbers J. Performance improvements for iterative electron tomography reconstruction using graphics processing units (GPUs). J. Struct. Biol. 176, 250–253 (2011). 21840398 10.1016/j.jsb.2011.07.017

