# Supplementary material for: Formation of bimetallic clusters in superfluid helium nanodroplets analysed by atomic resolution electron tomography
Source: Nat Commun. 2015 Oct 28;6:8779. doi: 10.1038/ncomms9779 (PMC4640115; doi:10.1038/ncomms9779)
Supplement: Supplementary Figures — 1-9 [file ncomms9779-s1.pdf]

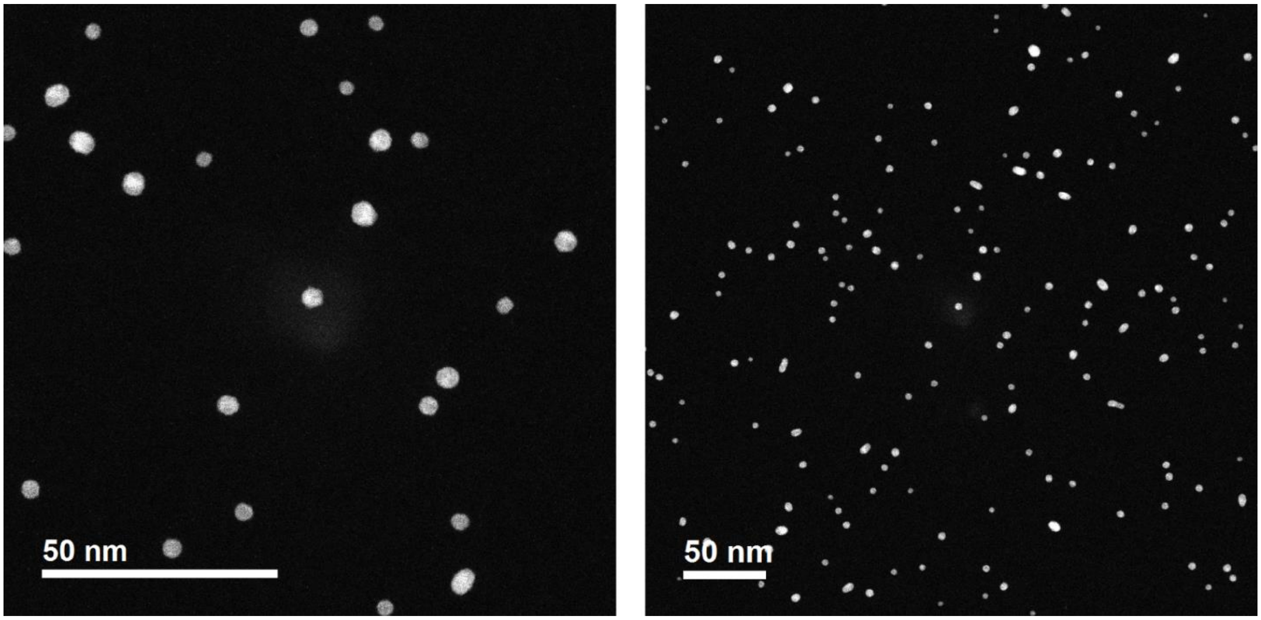

Supplementary Figure 1 | HAADF STEM overview images showing the distribution of clusters on the grid.

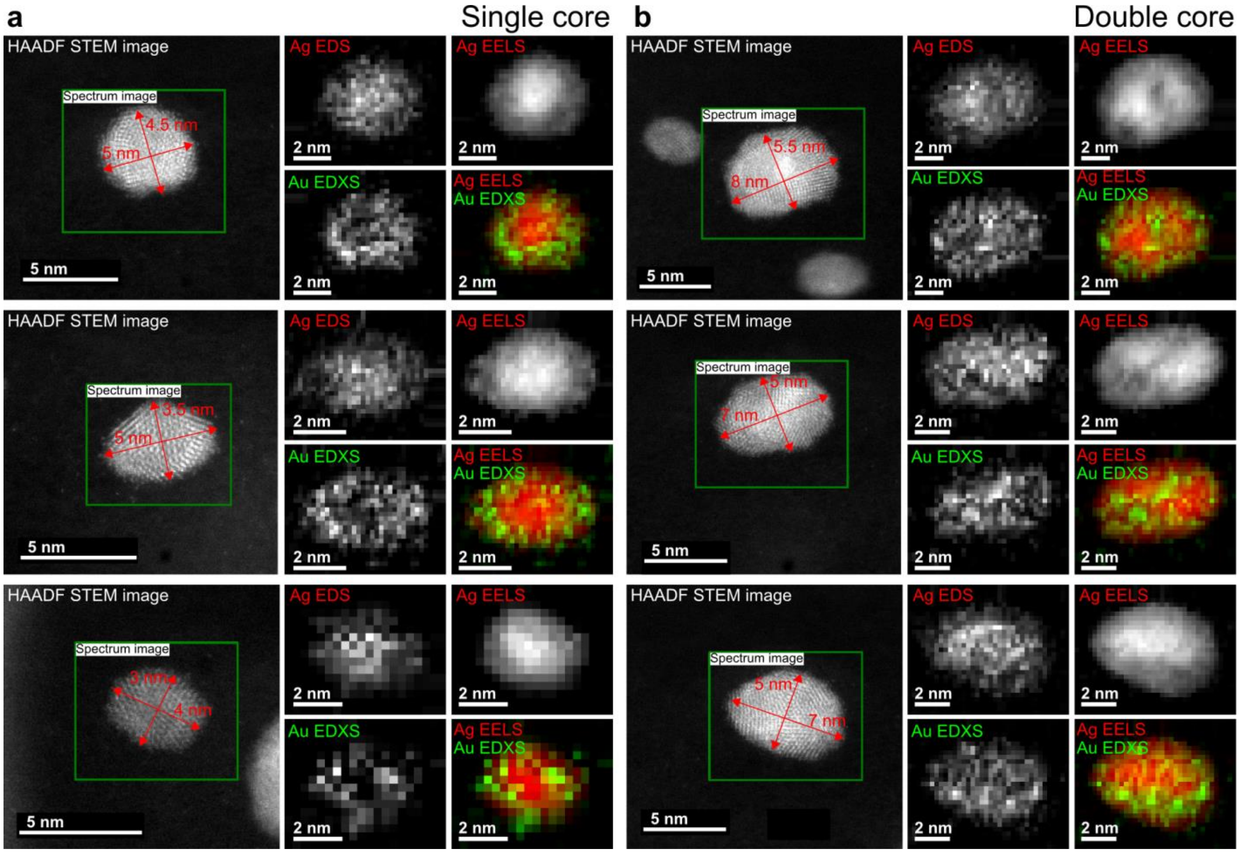

Supplementary Figure 2 | EELS and EDXS elemental maps of different (a) single and (b) double-core nanoclusters acquired at a tilt angle of 65°. The boxed region in the HAADF STEM image indicates the area of the mapped region and the size of the clusters. The maps have been generated from the EDXS signal of the Ag and Au L-lines, and for the EELS signal the Ag M-edge intensity was extracted. The color overlay was formed from the Ag EELS and the Au EDXS signal.

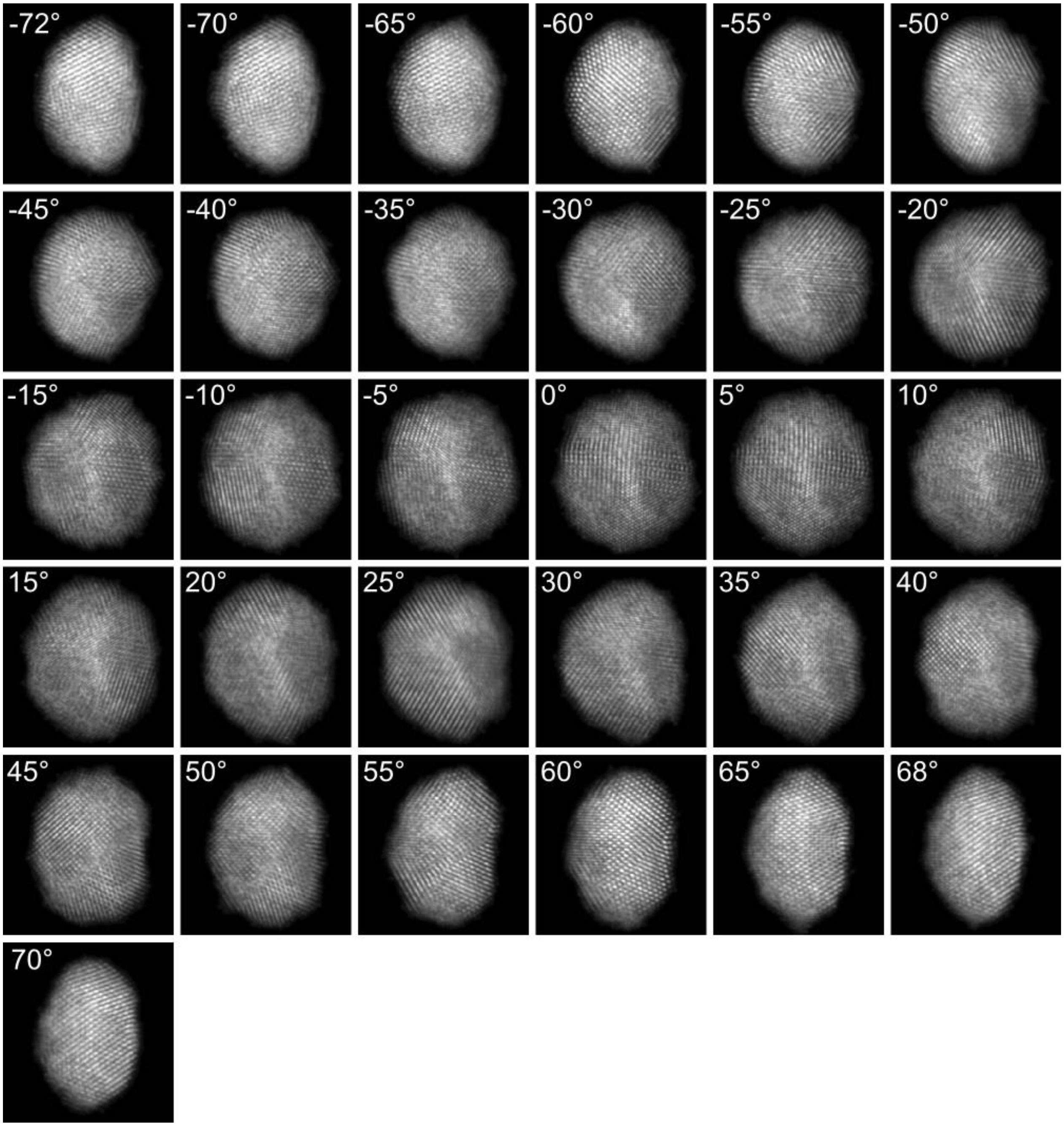

Supplementary Figure 3 | Projections of the tilt series. Each projection is the sum of 15 individual HAADF STEM images that have been aligned and corrected for drift.

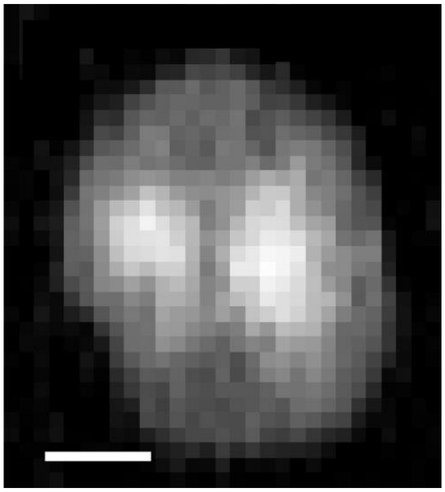

Supplementary Figure 4 | Ag M-edge EELS map of the double core nanoparticle at 0° tilt angle. Scale bar is 2 nm.

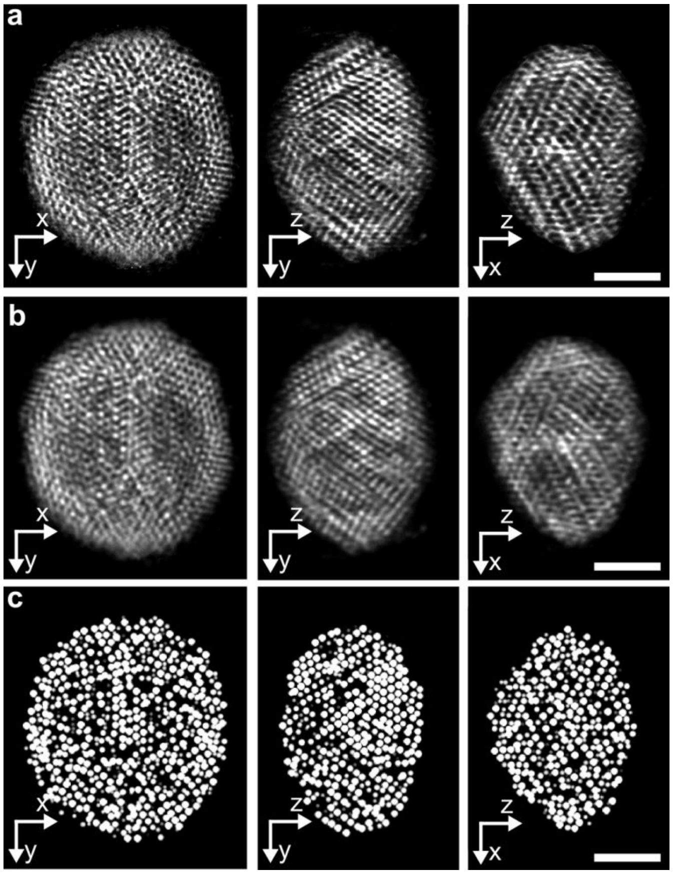

Supplementary Figure 5 | Detection of atomic positions: (a) slices through the original volume, (b) convolved with a Gaussian kernel. A local maximum search in (b) is used to estimate atomic positions, displayed as spheres in (c). Scale bars are 2 nm.

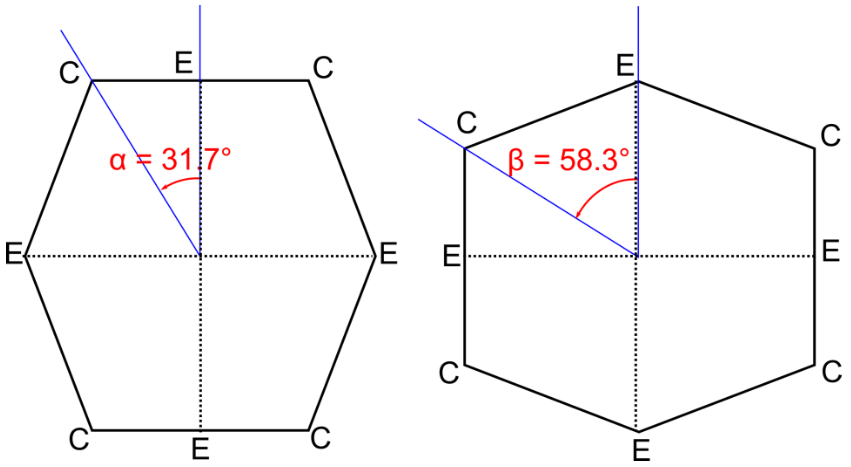

Supplementary Figure 6 | Rotation angles for centering five-fold symmetry axes as calculated based on the geometry of an ideal icosahedron. Corners (C) and edges (E) are indicated.

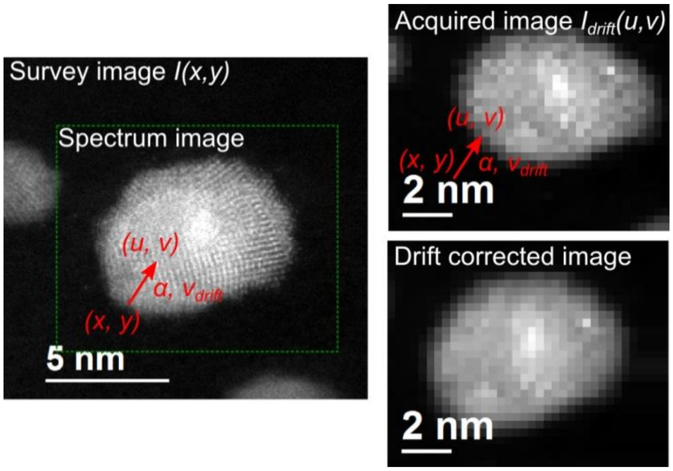

Supplementary Figure 7 | Drift correction of the spectrum images: a survey image *I(x,y),* recorded before the spectrum image acquisition, is used as a reference to calculate the drift direction α and the drift velocity v_{drift}) in the acquired image $I_{drift}(u,v)$. These parameters are subsequently used to correct for drift in the acquired image and all associated spectroscopic signals.

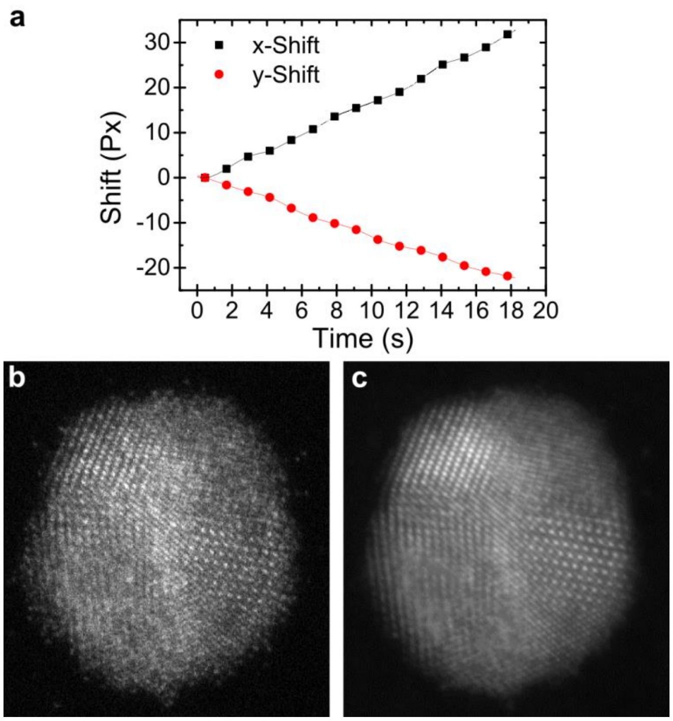

Supplementary Figure 8 | Drift correction and summation of HAADF STEM projections. (a) Drift in x- and y-direction as a function of time. Symbols denote the drift calculated for each image and used as drift value for the central pixel of each image. An interpolation over time is done to estimate the drift at each time instance during image acquisition. (b) is a single HAADF STEM image and (c) is the result of 15 drift corrected and aligned images.

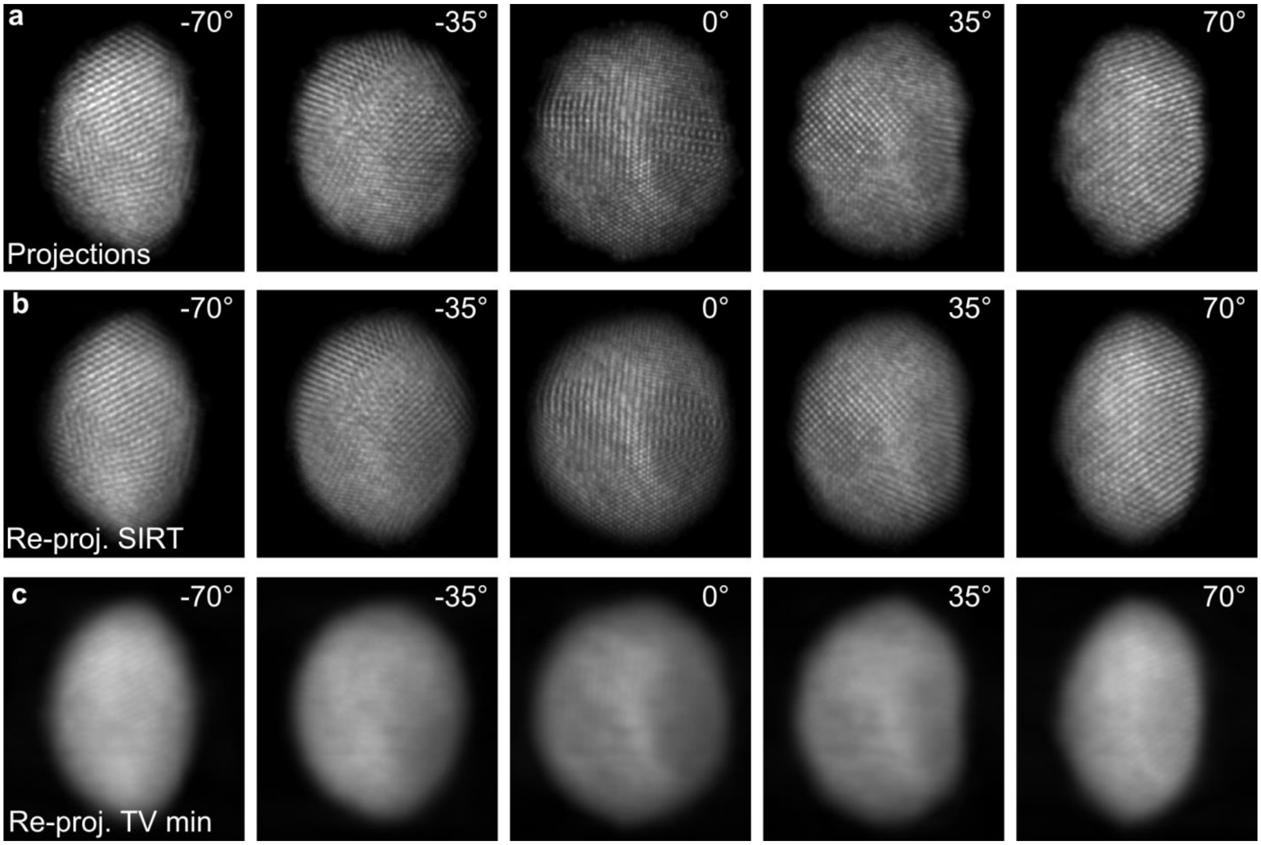

Supplementary Figure 9. Comparison of original projections and re-projections for five tilt angles: (a) summed projections as acquired, (b) SIRT re-projections (Fig. 2a), and (c) re-projections of the TV minimization reconstruction (Fig. 2b).
